# Dietary Patterns among Adolescents Are Associated with Growth, Socioeconomic Features, and Health-Related Behaviors

**DOI:** 10.3390/foods10123054

**Published:** 2021-12-08

**Authors:** Tali Sinai, Rachel Axelrod, Tal Shimony, Mona Boaz, Vered Kaufman-Shriqui

**Affiliations:** 1 Israel Center for Disease Control, State of Israel Ministry of Health, Ramat-Gan 5265601, Israel; tali.sinai@moh.gov.il (T.S.); axelrod1@mail.tau.ac.il (R.A.); Tal.Shimony@moh.health.gov.il (T.S.); 2 School of Nutritional Sciences, The Robert H. Smith Faculty of Agriculture, Food and Environment, The Hebrew University of Jerusalem, Rehovot 7610001, Israel; 3 Department of Nutrition Sciences, School of Health Sciences, Ariel University, Kiryat Hamada 3, Ariel 40700, Israel; monabo@ariel.ac.il

**Keywords:** adolescents, dietary habits, health-related behavior, obesity, height, nutrition survey, principal component analyses

## Abstract

Dietary patterns (DPs), usually established in adolescents, are important modifiable risk factors in the etiology of malnutrition and chronic diseases. This study aimed to identify DPs of adolescents and examine their associations with growth, sociodemographic, and lifestyle characteristics. A nationally representative, school-based, cross-sectional study was conducted in Israeli adolescents aged 11–18 years during 2015–2016. A self-administered survey queried sociodemographics, health behaviors, and diet. Weight and height were measured, and WHO height z-scores and BMI cutoffs were calculated. Food frequency questionnaire data were analyzed using principal components analysis (PCA) to identify DPs. Associations between growth, lifestyle, and sociodemographic characteristics and DPs were modeled using multivariable logistic regressions. A total of 3902 adolescents (46% males, mean age 15.2 ± 1.6 years) completed the survey. PCA identified five DPs, accounting for 38.3% of the total variance. The first two prominent DPs were the ‘plant-based food’ DP, which was associated with the female sex, higher socioeconomic status, overweight/obesity, and healthy lifestyle and the ‘junk food’ DP, which was associated with lower SES, unhealthy lifestyle, and lower height z-scores. Our results elucidate major DPs that strongly correlate with lifestyle risk behaviors and suboptimal growth among adolescents. Implementing screening for DPs should be further examined to identify higher risk health factors among youth.

## 1. Introduction

Adolescence is a critical period of development in which individuals acquire greater autonomy. This period is also characterized by accelerated physical growth and psychosocial and secondary sexual development. During this time, nutrition intake must be optimal to achieve full growth potential [1]. Along with the physiological changes, cognitive, emotional, and social changes occur, such as separation and individuation, which are expressed in adolescent eating behaviors such as social eating [2,3]. Dietary habits established during adolescence are likely to persist into adulthood; in addition, consuming a healthy diet in adolescence helps protect against malnutrition and noncommunicable diseases [4,5]. Prevention of obesity is especially important in light of the findings that approximately 80% of obese adolescents will remain obese in adulthood, and 70% will be obese at age 30 and beyond [6].

Biological, psychosocial, and cognitive changes throughout adolescence directly affect nutrition status and nutrient needs. The increased need for energy and nutrients among adolescents, combined with growing financial independence and the increasing need for autonomy when making food choices, coupled with immature cognitive abilities, place adolescents at risk for both over and undernutrition [3]. The food intake of adolescents is influenced by their peers, mass media, and body image. As a group, adolescents tend to snack and graze, skip meals, eat away from home, eat at late hours, and consume fast foods more frequently than younger children [7,8]. Other unhealthy behaviors, such as lack of physical activity, smoking, and drinking, often begin during adolescence and share a biological platform influencing and influenced by nutrition [9]. Increasing rates of overweight and obesity among children and adolescents and related diseases and eating disorders are of both individual and public health concern [10,11]. About 10% of Israeli adolescents are obese [12]. Poor dietary quality, measured by the adherence to the Mediterranean diet, was also documented in Israeli adolescents [13].

Diet is an important modifiable risk factor in the etiology of chronic diseases [4]. The study of dietary patterns (DPs) allows the examination of cluster food combinations unique to a population [14,15]. DPs identified in factor analysis may be more predictive of disease risk than an individual food or nutrient [15]. This methodology is intuitive because individuals consume foods rather than nutrients, and DPs represent patterns of food items [16]. Principal component analysis (PCA) represents the sum of the unweighted standardized food variables, which explain the greatest proportion of variability in a dietary pattern [17]. Further, this method permits the identification of clustering between DPs and other characteristics; for example, distinct dietary patterns have been shown to be influenced by socioeconomic and lifestyle characteristics, which contribute to the development of adolescent overweight and obesity [18,19].

The present study aims to identify DPs and associated health-related behaviors and socioeconomic factors among a representative sample of Israeli adolescents.

## 2. Materials and Methods

### 2.1. Population and Sampling

The study population, sampling method, and data sources in the 2nd Israeli Youth Health and Nutrition Survey (MABAT Youth 2) have been described elsewhere [20,21,22]. Briefly, the MABAT Youth Survey is a cross-sectional, nationally representative, school-based survey. A cluster sampling framework was used. Schools (7th–12th grades) were randomly selected from the Ministry of Education lists, ensuring representation of Israeli adolescents according to school level (middle school: grades 7–9; high school: grades 10–12), primary language spoken at home (Hebrew or Arabic speakers), and school welfare level. Schools that are not under the supervision of the Israeli Ministry of Education (students from the ultra-orthodox sector, private or boarding schools), were not included in the sample [20]. The school compliance rate was high; 217 schools participated out of 234 schools approached for inclusion (92.7%). Of the 7029 possible participants, 5589 students were surveyed (79.5%), largely due to nonattendance on the day of the survey.

The MABAT Youth 2 survey was reviewed and approved by the Ethics Committee of the Sheba Medical Center. According to the Israeli Ministry of Education guidelines, all parents received written information regarding the survey. Only students whose parents did not oppose writing the participation of their children were included in the study. The students reserved the right to refuse participation in any part of the survey. Participating students completed a self-administered questionnaire, which included sociodemographic information, health behaviors, and a food frequency questionnaire (FFQ). The questionnaires have been described in detail elsewhere [20,22].

The present analyses included 3902 (69.8%) students who completed the FFQ (Supplementary Materials Figure S1). Lower FFQ completion rates were found among boys (72.4% vs. 80.8% among girls), Arabic speakers (62.6% vs. 81.2% among Hebrew speakers), and those from lower SES backgrounds (65.4% vs. 80.1% among moderate to high SES).

### 2.2. Definition of SES

Socioeconomic status was determined using Welfare Index rank of financial support required by the school, as identified by the Israel Ministry of Education [21,22,23]. Welfare indices are on the basis of family income, parental education, immigration status (from underdeveloped or developed countries), and school location. A score is calculated for each individual student and the Welfare Index for a specific school is the average of scores of the registered students. For this study, low SES was defined as any school ranked from 7 to 10. All other schools (ranked 1–6) were considered moderate to high SES, as previously reported [21].

### 2.3. Anthropometric Measurements

Anthropometric measurements, including height and weight, were performed using standardized measurement tools and trained research assistants [20]. Body mass index (BMI) was calculated as weight in kilograms divided by height in meters squared (weight [kg]/height [m2]). The World Health Organization (WHO) growth standards were used to calculate height and BMI z-score values according to children’s age, and sex. BMI z-scores were divided into four weight categories: “underweight” less than −2 SD, “normal weight” ≥ (−2)–1 SD, “overweight” ≥ 1–2 SD, and “obese” ≥ 2 SD [24].

### 2.4. Health-Related Behaviors

Health-related behaviors reported in the study questionnaire were defined using a series of direct questions, which also provided internal construct validity [20].

Smoking (yes/no) was defined as “yes” for participants who reported any current cigarette and/or hookah smoking. Consumption of alcoholic beverages was recorded (type, frequency, and amount), and alcohol drinking was defined as “yes” for those who drank at least one serving of alcoholic beverages per month. Physical activity (PA) was also recorded (type, frequency, and amount). Participants who performed any PA (walking, cycling, running, swimming, ball games, etc.) for at least an hour a day, on average, were defined as ‘yes’ for performing physical activities as recommended [25]; others were identified as ‘no.’ Fast food intake was defined as “low” for participants who reported eating fast food (e.g., hamburgers, falafels, pizza) zero to three times a week, and “high” for those who consumed these foods four times/week or more [13,20].

### 2.5. Dietary Assessment

The habitual dietary intake of participants was estimated using a semiquantitative food frequency questionnaire (FFQ), which has been described in detail elsewhere [13,20,22]. This questionnaire is based on validated FFQ developed to assess the dietary intake of Israeli multiethnic populations [26,27]. The questionnaire was assessed by a national steering committee, which adopted it for adolescents. The adopted questionnaire has been used in the first and second Israeli MABAT Youth surveys [13,20]. Briefly, the questionnaire includes a 103 item food list, with a standard portion size provided for each food item. For all items, the questionnaire enables reporting the quantity usually eaten by adding the number of portions eaten in an open column, presenting four frequency options: “number of portions a day/a week/a month” or “never or less than once a month”. Self-administered, the FFQ was completed after participants received instructions from study personnel. Additionally, a sub-sample of 467 (12%) students was interviewed using a 24 h food recall to calibrate the macronutrient values generated from the FFQ [20].

### 2.6. Food Component Derivation

We used principal component analysis (PCA), a method frequently used in nutrition research, to identify dietary patterns (DPs) for inclusion in statistical models [14,28]. PCA captures the total food item variance explained by the selected variables, with factor loadings representing the correlation between the variable and the factor. Food items in the FFQ were grouped by three expert dietitians, into 37 food groups, according to similarities in nutrients (e.g., grains, fruits, and desserts); the food groups and related foods are presented in Supplementary Materials Table S1. The mean energy contributed by a food group per day was calculated, and a variable representing it as a percentage from the daily energy was derived. The PCA was performed with orthogonal rotation with varimax option to derive optimal distinctive dietary patterns [29]. The correlation matrix of the variables was examined to decide the number of components to retain based on eigenvalue and interpretability. Food groups with an absolute factor loading greater or equal to 0.35 were used to characterize the dietary patterns [15,19].

### 2.7. Statistical Analyses

Statistical analyses were performed using SAS version 9.4. Differences between the independent variables were examined in univariate analyses using the Mann-Whitney U test for continuous variables and the Chi-square test (χ^2^) for categorical variables. These descriptive statistics were calculated using sampling weights (performed according to sex, primary language, and school level). Quartile cut points (Q1–Q4) were calculated from the continuous DPs for all food patterns, with Q1 being the lowest consumption of the DP and Q4 being the highest consumption of the DP. A dichotomized outcome component was created, comparing the highest quartile (Q4) to all other quartiles (Q1–Q3). Associations between height z-scores, weight status, and selected socioeconomic factors, and health behaviors that were associated with the outcome variable at a level of *p* < 0.10 in univariate analysis were considered for inclusion in the multivariable analysis. Adolescents who were underweight (*n* = 70) were omitted from the regression model to allow the dichotomization of the weight status to overweight/obese vs. normal. The covariates that met this criterion were tested for multicollinearity. Multivariable logistic regression models were then fitted to test the association between the potential sociodemographic variables and health behaviors and DPs. Odds ratios and confidence intervals (95% CI) are presented. All tests were two-tailed and considered significant at *p* < 0.05.

## 3. Results

In total, 3902 adolescents in grades 7–12, aged 11–18 years, completed the self-administered questionnaire, including the FFQ, eating habits, and lifestyle questionnaires. Socioeconomic, health-related behaviors, and food intake data are presented in Table 1. A few lifestyle outcomes and anthropometric measures were complete for about 90% of our participants. However, we noted no differences in socioeconomic background between individuals with complete vs. missing information. The majority of participants were normal weight, though 30% were overweight or obese. Total energy consumption (Mean ± SD) was 2316 ± 1083 Kcal. As percentages of energy intake, macronutrient values were within the acceptable macronutrient distribution ranges according to the DRI recommendations [30]: 54.4% of the energy was from carbohydrates, 12.2% from proteins, and 31.1% from fat. Significant differences were noted by gender. Slightly higher percentages of males spoke Hebrew as their first language, while more females were of low SES. A higher proportion of females were of normal weight. Compared to males, the prevalence of smoking, alcohol intake, and fast food intake was lower in 40–50%. Energy consumption was higher in males, and so were the percentages of carbohydrates and protein from total energy.

### 3.1. Generation of Indices

In the first stage of the PCA, a total of 15 factors representing food patterns were generated, capturing 53.7% of the variance. Based on the a priori rule, variables that loaded lower than 0.35 on each of the factors were not entered into the second stage. PCA performed on the remaining variables generated five final DPs capturing unique food patterns. Factor loadings are presented in Supplementary Materials Table S2. Five DPs accounted for 38.3% of the total variance, the first DP accounted for 10.5% of the total variance, and the remaining DPs 2–5 added 8.0%, 7.1%, 6.4%, and 6.3% to the total variance, respectively. Food groups loaded either positively or negatively on each of the DPs. Specifically, a positive loading indicated a high intake of the item within the specified DP, while a negative loading indicated a low intake of the item.

### 3.2. Dietary Patterns

The first DP, ‘plant-based food’, was characterized by positive loadings on fresh and cooked vegetables, fruits, and legumes (by descending order of PCA loading) and negative loading of white bread.

The second DP, ‘junk food’, was positively associated with soft drinks, salty snacks, cream cakes, and ice cream and was negatively associated with white bread and eggs. The third DP, ‘hot sweetened beverage and spreads,’ was characterized by positive loadings of sugar, honey, jam, coffee and tea, high fat spreads (butter, margarine, and mayonnaise), and oils (avocado, olive oil, and canola oil). The fourth DP, ‘cereals and milk’, loaded all breakfast cereals, milk, and dairy chocolate drinks and puddings positively and negatively for white bread. The fifth DP, ‘carnivore’, was positively associated with beef and chicken, meatballs, hamburgers, schnitzel, and kabab and negatively associated with the consumption of white bread.

### 3.3. Dietary Patterns and Associated Growth, Demographic, and Health-Related Characteristics

Crude associations between the highest quartile of each DP (Q4) in comparison to all other quartiles (Q1–Q3) to growth, demographic, and health-related characteristics were calculated. Differences in adherence to all five DPs were highly and diversely associated with most sociodemographic and health factors. Odds ratios were calculated to examine these associations in multivariable analyses (Table 2). The following associations were significant: the first DP, ‘plant-based diet’, correlated highly with students who were the female sex, Arabic speakers, from higher socioeconomic backgrounds, attending either middle or high school, having increased height z-scores, and being overweight/obese. Males were 68% less likely to be in the highest quartile (in comparison to Q1–Q3) of a plant-based diet, those who consumed fast food four times a week were 73% less likely to be in the highest quartile of a plant-based diet, and those who adhered to the recommendations for PA were 55% more likely to be in the highest quartile of a plant-based diet.

The second DP, ‘junk food’, correlated highly with Arabic speakers attending middle school and students identified as low SES. This DP was inversely associated with stature. Every decrease of 1.0 SD in height z-scores was associated with an increase of 91% odds to be in the highest quartile of this DP. In comparison to nonsmokers, those who smoked had 78% higher odds to be in the highest quartile of junk food consumption. Additionally, those who reported eating fast food four times a week or more in comparison to three times and below were 2.2 times more likely to be in the highest quartile of junk food DP.

The third DP, ‘hot sweetened beverages and spreads’, correlated highly with high school grades attending and Hebrew-speaking students. Those who smoked, in comparison to nonsmokers, were 43% more likely to be in the highest quartile of this DP. Adherence to PA guidelines was associated with a decrease of 23% to be in the highest quartile of the DP, and those who drank alcohol at least once a month were 56% more likely to be in the highest quartile.

The fourth DP, ‘cereals and milk’, correlated with males and Hebrew speakers. Those who ate fast food four times per week or more were 45% less likely to be in the highest quartile of this DP. Students who adhered to the recommendations for PA were 18% more likely to be in the highest quartile of this DP.

Hebrew speakers were 2.5 times more likely to be in the highest quartile of the DP ‘carnivore’. Being of normal weight decreased the odds of being in the highest quartile of ‘carnivores’ by 27%. Those who consumed fast food four times per week or more were 82% more likely to be in the highest quartile of ‘carnivores’, and those who adhered to the recommendations for PA were 29% more likely to be in the highest quartile of this DP. Additionally, the male sex increased the odds by 40%. The negative association to SES was not statistically significant (*p* = 0.09).

## 4. Discussion

In this representative sample of 3902 Israeli adolescents who reported their dietary intake using a detailed FFQ, mean macronutrients, as percentages of energy intake, were within the acceptable macronutrient distribution ranges [30]. We identified five unique DPs and detected significant associations between those DPs and sociodemographic and health-related behaviors and characteristics. The five major DPs were: ‘plant-based food’, ‘junk-food‘, hot sweetened beverages and spreads’, ‘cereals and milk’, and ‘carnivore’. All dietary patterns identified in this study are associated with specific sociodemographic characteristics.

In this population, the ‘plant-based food’ DP showed the highest percentage of dietary variance. In a study among Israeli adolescents, healthier eating was characterized by a higher adherence to the Mediterranean diet pattern, including a high intake of vegetables, fruits, and legumes [31]. In agreement with our findings, this pattern characterized adolescents from higher SES and Arabic speakers. Interestingly, this healthy DP was also associated with a higher frequency of physical activity and a lower frequency of fast food intake; counterintuitively, it was positively associated with overweight/obesity. Thus, this DP may capture adolescent girls who are more aware of healthy lifestyle behaviors and try to maintain healthy body weights. Similar findings were described among overweight and obese Hispanic adolescent girls from Los Angeles who expressed higher motivation and competence for eating healthier than their peers [32]. A positive association between higher adherence to similar healthy dietary patterns named ‘natural’/’prudent’ and overweightness was also reported in Chilean [19] and Mexican [14] adolescents. Increased dietary quality scores and higher micronutrient consumption were recently reported among overweight and obese school children and adolescents from Iran [33].

The ‘cereal and milk’ DP was characterized by high consumption of breakfast cereals, milk, and chocolate milk associated with Hebrew speakers and the male sex, and lower odds for frequent fast food intake. Consumption of breakfast cereals and milk among adolescents has been found to be associated with lower body fatness among Scandinavian adolescents [34] and was associated with better overall nutrition among Irish adolescents [35], although some cereals are defined as ultra-processed foods, which are generally considered unhealthy [36].

Unhealthy DPs have been associated with adverse health outcomes. A recent review showed a positive association between unhealthy dietary patterns and cardiometabolic risks in children and adolescents [37]. Among adolescents in Mexico, ‘Western’ and ‘high protein and fat’ DPs were associated with higher BMI [14]. In our data, the ‘junk-food’ DP was associated with soft drinks, salty snacks, and ice cream and negatively with bread and eggs. This pattern is high in fat, sugar, and sodium and has reduced nutritional value/quality. This pattern was associated with Arabic speakers, lower SES, smoking, and lack of physical activity, together with increased odds of frequently consuming fast food. In the current study, this DP was inversely associated with stature, which indicates long term, inadequate nutritional intake [38]. Increased diet variability explained by this pattern is of concern because adolescence is a critical period for rapid growth and development. These eating behaviors may result in suboptimal growth and lead to adverse health consequences in later years [39]. Efforts are needed to promote adolescents’ use of the new front-of-package warning labels implemented by the Israeli Ministry of Health and identify products with high sodium, sugar, and saturated fats [40].

The ‘hot sweetened beverages and spreads’ DP was consistent with fast food consumption and high sugar and fats intake. This DP was associated with Hebrew speakers, the female sex, smoking, drinking alcohol, and inadequate physical activity. This DP is consistent with the findings in a national school-based survey of Brazilian adolescents [41]. Drinking coffee, sugar-sweetened beverages, and high energy drink consumption were associated with increased high risk behaviors, including smoking as adults [42]. In a representative sample of Canadian adolescents, high energy drink consumption on four or five days of the school week was the best predictor of smoking behavior [43]. High consumption of warm, sugar-sweetened beverages was also reported among Korean adolescents [44].

The fifth dietary pattern, ‘carnivore’, is characterized by high intake of beef, poultry, and processed meat products and low bread intake. This DP is characterized by the male sex, overweight/obesity, frequent physical activity, and frequent fast food intake. The association between the consumption of processed meat products and adverse cardiometabolic outcomes is well-established among adults [45,46] and adolescents [47]. The association between excess body weight and meat eating may add to those risk factors in the present study population. The association between obesity and increased physical activity, identified herein, has also been detected in a previous study [48]. In addition, male students in this study were more likely to have DPs that included animal protein, ‘cereals and milk’, and ‘carnivore’ DPs, which are also associated positively with exercising. Consuming more protein while exercising is a common approach used for reducing fat mass and maintaining/building muscle mass [49].

No consistent relationship was found between dietary patterns and age groups (school level) in the present study. The ‘junk food’ pattern was found to be associated with middle school age, while the snacking pattern of high sugar and fats was found to be more related to high school age. Adolescents participating in the multinational Health Behaviors in Schools study demonstrated higher sedentarism and a lower intake of fruits and vegetables with age increases [50], suggesting that unhealthy eating behaviors may increase with age. Generally, eating patterns that represent a healthier diet (i.e., with characteristics of the Mediterranean diet recommended by the Israeli Ministry of Health [51]), for example, the plant-based DP, appear to be associated with health-promoting behaviors and high socioeconomic status, while others, such as the ‘junk food’ and ‘carnivore’ DPs, were related with unhealthy behaviors and low socioeconomic backgrounds. The association between lower SES and unhealthy DPs identified in the present study has also been reported in a scoping review among adolescents from lower SES [52]. Consistent with this, the co-occurrence of unhealthy eating, smoking, alcohol abuse, and insufficient physical activity has been described among a national sample of Brazilian adolescents [40].

Our study has some limitations; the first limitation is that the PCA procedure is a data-driven methodology that may be influenced by the way food items were grouped [53]. Another possible limitation is self-reported information. Food intake and physical activity, for example, may be influenced by social desirability bias. Nevertheless, most surveys use self-administered questionnaires; the risk of bias is reduced by assuring respondent anonymity. Despite a difference between the total population and the population which replied to the FFQ and presented herein, our results are consistent with outcomes observed in other reports. Additionally, our study was limited to several health outcomes due to the nature of the original MABAT Youth survey. Therefore, it is mainly confirmative. Finally, because the study uses a cross-sectional design, causality cannot be inferred.

The strengths of this research include the use of data from a large, national, representative sample of youth in Israel, which reflects the ethnic diversity of the population. The large sample permitted a robust evaluation of health behaviors and dietary intake using a validated FFQ. Second, anthropometric measurements were carried out by trained study personnel, which eliminated misclassification. An additional strength is the use of SES classifications based on the Israeli Ministry of Education, which prevented inaccuracies of self-reported SES by the adolescent respondents.

## 5. Conclusions

Our findings characterize five eating patterns, each associated with SES, lifestyle, growth, and risk behaviors among Israeli adolescents. Findings of the present study suggest that screening for DPs may serve as a surrogate for other high risk health factors. Of course, the sensitivity of nutrition screening for general health risks must be directly tested. If proven accurate, DP screenings could conceivably assist policymakers in designing preventive interventions and should be further researched. In addition, efforts should be made to include students attending private/ultra-Orthodox schools in future research.

## Figures and Tables

**Table 1:** Selected sociodemographic, dietary intake and health-related characteristics of the study participants by sex ^a^.

Characteristics	*n*	All *n* = 3902	Male *n* = 1786	Female *n* = 2116	*p* ^b^
Demographic Data					
Age (years)	3902	15.2 ± 1.6	15.3 ± 1.7	15.2 ± 1.5	0.49
School (% high schools)	3902	1717 (49.1%)	766 (49.4%)	951 (48.8%)	0.20
First language (% Hebrew)	3902	2741 (79.6%)	1339 (80.8%)	1402 (78.4%)	<0.0001
Socioeconomic status (% low)	3902	1394 (34.2%)	601 (33.1%)	793 (35.2%)	0.013
Health-Related Characteristics					
Height z-score	3530	−0.06 ± 1.02	0.04 ± 1.08	−0.17 ± 0.96	<0.0001
Body mass index categories	3500				0.036
Underweight	70	70 (2%)	34 (2.1%)	36 (1.9%)	
Normal weight	2366	2366 (68.3%)	1044 (66.3%)	1322 (70.1%)	
Overweight/obesity	1064	1064 (29.7%)	519 (31.6%)	545 (28.0%)	
Physical activity (≥1 h a day)	3493	1226 (34.8%)	768 (47.5%)	458 (23.4%)	<0.0001
Smoking (%)	3736	299 (9.3%)	185 (12.4%)	114 (6.6%)	<0.0001
Alcohol intake (≥1 beverage a month)	3692	583 (18.1%)	325 (22.5%)	258 (14.2%)	<0.0001
Eating fast food (≥4 times a week)	3853	164 (4.0%)	96 (5.3%)	68 (2.9%)	<0.001
Dietary Intake					
Energy (Kcal/d)	3902	2316 ± 1083	2426 ± 1118	2214 ± 1043	<0.0001
Total fat (% Energy)	3902	31.1 ± 4.9	31.2 ± 4.9	31.1 ± 4.9	0.74
Total carbohydrates (% Energy)	3902	54.4 ± 7.1	53.9 ± 7.2	54.8 ± 7.0	<0.0001
Total proteins (% Energy)	3902	12.2 ± 2.6	12.5 ± 2.7	11.8 ± 2.4	<0.0001

^a^ Calculated with the application of sample weights of the Israeli Youth Health and Nutrition Survey. Categorical variables are expressed as *n* (%), continuous variables are expressed as mean ± SD. ^b^ Significance is derived from Mann-Whitney U test for continuous variables and Chi-square test (χ^2^) for categorical variables.

**Table 2:** Adjusted odds ratios (with 95% confidence intervals) comparing Quartiles 1–3 with Quartile 4 (highest) for each dietary pattern with selected sociodemographic and health-related variables. Results from multivariable logistic regression analyses ^1^.

Variables	Plant-Based Food		Cereals and Milk		Junk Food		Hot Sweetened Beverages and Spreads		Carnivores	
	OR (95% CI)	*p*	OR (95% CI)	*p*	OR (95% CI)	*p*	OR (95% CI)	*p*	OR (95% CI)	*p*
Male vs. female	0.32 (0.26–0.38)	<0.0001	1.19 )1.00–1.42)	0.05	0.92 (0.76–1.11)	0.40	0.63 (0.52–0.75)	<0.0001	1.40 (1.17–1.67)	<0.001
Attending middle school class vs. high school class	0.96 (0.79–1.16)	0.66	1.13 (0.94–1.37)	0.19	1.50 (1.23–1.83)	<0.0001	0.55 (0.46–0.67)	<0.0001	0.94 (0.77–1.13)	0.49
Hebrew speakers vs. Arabic speakers	0.68 (0.56–0.83)	<0.0001	1.55 (1.26–1.91)	<0.0001	0.49 (0.41–0.60)	<0.0001	1.98 (1.60–2.46)	<0.0001	2.47 (1.97–3.09)	<0.0001
Middle–high SES vs. low SES	1.50 (1.23–1.82)	<0.0001	1.10 (0.91–1.33)	0.31	0.48 (0.40–0.57)	<0.0001	1.04 (0.86–1.26)	0.66	0.85 (0.71–1.03)	0.09
Height z-score (SD)	1.15 (1.05–1.25)	0.002	0.99 (0.91–1.08)	0.78	0.91 (0.83–0.99)	0.04	1.07 (0.98–1.16)	0.16	0.94 (0.86–1.02)	0.15
Normal weight vs. overweight and obese	0.65 (0.54–0.78)	<0.0001	0.86 (0.71–1.03)	0.10	1.14 (0.94–1.39)	0.20	0.84 (0.70–1.02)	0.08	0.73 (0.61–0.88)	0.001
Smoking vs. non-smoking	0.97 (0.67–1.41)	0.87	0.94 (0.66–1.34)	0.73	1.78 (1.27–2.51)	0.001	1.43 (1.04–1.98)	0.03	1.19 (0.85–1.67)	0.30
Physical activity as recommended vs. less	1.55 (1.28–1.87)	<0.0001	1.18 (0.98–1.41)	0.08	0.84 (0.69–1.03)	0.09	0.77 (0.63–0.93)	0.008	1.29 (1.07–1.55)	0.008
Alcohol drink at least once a month vs. less	1.03 (0.80–1.34)	0.80	0.98 (0.76–1.26)	0.86	1.30 (0.99–1.70)	0.06	1.56 (1.23–1.98)	0.0003	1.09 (0.85–1.40)	0.52
Fast food consumed 4 times a week or more vs. 3 or fewer	0.27 (0.14–0.53)	<0.001	0.55 (0.32–0.94)	0.030	2.20 (1.48–3.28)	<0.001	0.83 (0.52–1.35)	0.46	1.82 (1.20–2.77)	0.005

CI, confidence interval; SES, socioeconomic status. ^1^ Adherence to each dietary pattern (Quartile 4 vs. Quartiles 1–3) adjusted to sex, school level, weight status, height z-score, SES, first language, smoking, physical activity, drinking, fast food intake.

## Data Availability

Data will be provided upon request and in accordance with the decision of the ICDC’s Publications Committee.

## Supplementary Materials

The following are available online at https://www.mdpi.com/article/10.3390/foods10123054/s1, Figure S1: Flowchart of study participants, Table S1: Thirty-seven food groups, which were driven from the original Food Frequency Questionnaire, Table S2: Varimax-rotated factor loadings of the five dietary patterns.
