# Supplementary material for: Dietary Patterns among Adolescents Are Associated with Growth, Socioeconomic Features, and Health-Related Behaviors
Source: Foods. 2021 Dec 8;10(12):3054. doi: 10.3390/foods10123054 (PMC8700870; doi:10.3390/foods10123054)
Supplement: Supplementary file 1 [file foods-10-03054-s001.zip › foods-1478384-supplementary.pdf]

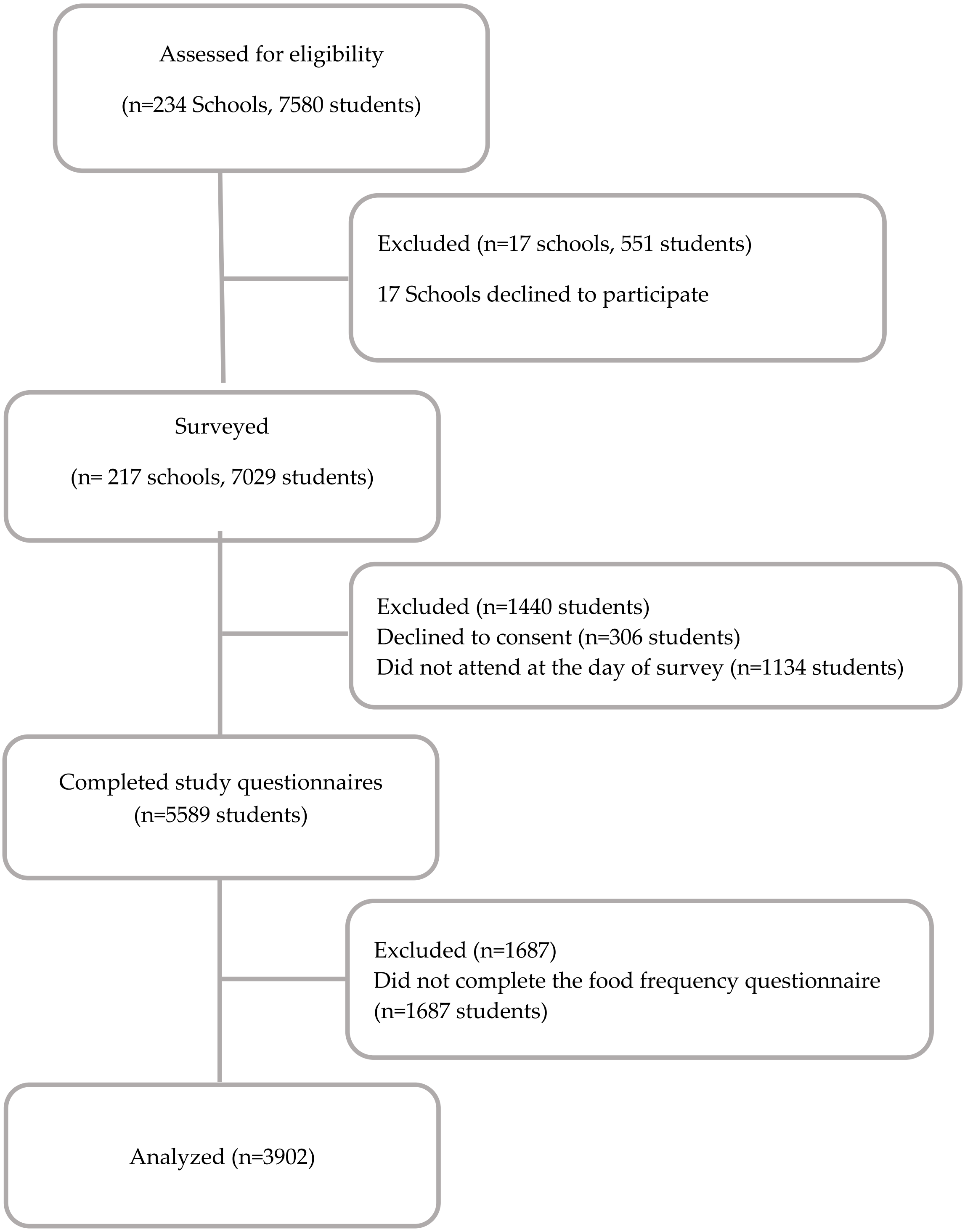

Figure S1. Flowchart of study participants

Supplemental Table S1

Thirty -seven food groups which were driven from the original Food Frequency Questionnaire

**Group code**	**Group name**	**Food item/Related foods**
0	Water	Water
1	Milk	Cow's milk (irrespective of fat %)
2	Bread	Bread, whole wheat or rye including toast
		Light/ diet bread (all types) including toast
		Injera (traditional Ethiopian bread)
		Tabun bread (traditional flat Arab bread)
		Pita with Za'atar (traditional Arab bread with spices)
		Pita bread (traditional Arab bread)
3	Grains	Rice, cooked
		Pasta, spaghetti, noodles, short-cut pasta
		Couscous with or without gravy
4	Cereals	Unsweetened breakfast cereals, e.g., cornflakes or bran flakes
		Sweetened breakfast cereals, e.g., Frosties or Coco pops
		Cream-filled Breakfast cereals e.g., Fillows
5	Cakes	Cookies
		Plain cake, e.g., sponge, chiffon or pound cake
		Individual pastry or danish (e.g., croissant)
6	Fruits	Apple, pear
		Citrus fruit e.g. orange, tangerine
		Banana
		Plums, peaches, nectarines
		Melon
		Watermelon
		Other fruit, e.g., persimmon, mango, guava, strawberries, grapes
7	Starchy vegetables	Potatoes, mashed
		Potatoes, cooked or baked in the oven
		Corn, Sweet corn
8	Cooked vegetables	Green beans, cooked
		Zucchini or eggplant baked or cooked
		Carrot, cooked or raw
		Broccoli or cauliflower
		Other cooked vegetables (e.g. peas, mushrooms, pumpkin, spinach)
		Vegetable soup
9	Fresh vegetables	Fresh vegetable salad
		Cabbage salad
		Tomatoes including cherry tomato (including in a sandwich, not including salad)

		Cucumber (including in a sandwich, not including in salad)
		Peppers- all colors
		Other fresh vegetables eg: kohlrabi, fennel
10	Legumes	Cooked legumes e,g, chickpeas, white beans, lentils
11	Sugars	Sugar (added to drinks/ cereals/yogurt, etc.)
		Honey or jam
12	Candies & sweet drinks	Popsicle
		Candies- any kind
		Carbonated sugar-sweetened drink
		Energy drink (XL or BLU)
		Sweet soft drink such as Spring or water with syrup
		Flavored water
13	Fruit juice	Fruit juice drink/ nectar (orange, apple, banana, strawberry, or other)
14	Chocolate	Chocolate spread
		Chocolate (squares)
		Chocolate bar (e.g., Mars Bar, Maltesers, Kit Kat)
		Cereal snack, Energy bar, Shugi, Nature-valley,
15	Fats (MUFA)	Avocado (including avocado salad)
		Olive or canola oil (not including the oil in a salad)
16	Sugar-free drinks	Diet carbonated soft drink (e.g., Diet Coke, diet Sprite, etc.
17	Salty snacks	Crackers, any kind
		Salty snacks- e.g., crisps, Doritos (Tortilla chip crisp), Cheetos (cheese snack), Bamba (peanut butter flavored snack), pretzels
18	Yogurt & soft cheese	Natural Yogurt (e.g., Leben, Kafir or other unsweetened yogurts)
		Yogurt with fruit or other toppings (granola, chocolate)
		Soft cheese (including additional ingredients, e.g., olives)
		Cottage cheese (including additional ingredients, e.g., olives)
19	Hard cheese and high-fat dairy	Sour cream
		Hard cheese, any kind
		Salty cheese, e.g., Bulgarian cheese
20	Chocolate drink & deserts	Chocolate drink (based on milk)
		Chocolate/vanilla pudding
		Ice cream
21	Oily spreads	Margarine, butter
		Mayonnaise, any type including salad dressing
22	Beef & chicken	Beef, steak, roast
		Chicken
23	Processed beef	Schnitzel (chicken/turkey)
		Meatballs: beef, chicken, turkey
		Hamburger or kabab
		Salami, pastrami
		Sausages (hot dogs)
24	Fish	Fish, fried or baked
		Tuna (in oil/ water)

25	Egg	Egg, fried, omelet, scrambled or egg dishes (e.g., 'Shakshuka')
		Egg, soft/hard-boiled
26		Vegetarian schnitzel/sausage (including corn schnitzel)
27	Hummus & Tahini	Hummus salad
		Tahini paste (traditional sesame paste)
28	Nuts & seeds	Seeds (sunflower, pumpkin, watermelon)
		Walnuts, almonds, pistachios, peanuts
29	Fried foods	Falafel
		French fries
30	Savory Pastries	Bourekas
		Melawach, Jahnun (traditional Yammaite pasteries)
31	Pizza	Pizza
32	Pickles	Olives
		Pickled vegetables (e.g.,cucumber, eggplant, tomato)
33	Alcohol drinks	Alcoholic beer
		Wine
34	Hot drinks	Tea - any kind
		Coffee (including with milk)
35	Deserts	Cream cake or cheesecake
		Ice cream popsicle (dairy)
36	White bread	White bread, challah including toast
		White bun (roll)
		Pita
37	Ketchup	Ketchup

Food items were grouped from an original list of 103 food items in the Mabat-Youth 2 food frequency questionnaire (FFQ).

Supplemental Table S2

Varimax-rotated factor loadings of the five dietary patterns

**Loadings for the first component: ''plant-based food'**	
**Variables**	**Principal component loading**
Vegetables, fresh	0.68
Vegetables, cooked	0.65
Fruit, fresh	0.64
Legumes	0.47
White bread	-0.47
**Loadings for the second component: 'junk-food'**	
**Variables**	**Principal component loading**
Candies and soft drinks	0.57
Salty snacks	0.55
Cream cakes and ice cream	0.55
White bread	-0.45
Eggs	-0.36
**Loadings for the third component:' hot sweetened beverages and spreads'**	
**Variables**	**Principal component loading**
Sugar, honey, or jam	0.64
Coffee and Tea	0.64
Butter, Margarine, and Mayonnaise	0.46
Avocado, Olive oil, and Canola oil	0.39
**Loadings for the fourth component: 'cereals and milk'**	
**Variables**	**Principal component loading**

Milk	0.66
Breakfast cereals, all types	0.59
Chocolate drinks and pudding	0.35
White Bread	-0.36
**Loadings for the fifth component: 'carnivores'**	
**Variables**	**Principal component loading**
Beef and Chicken	0.69
Meatballs, Hamburgers, Schnitzel, Kabab	0.57
White bread	-0.42
